# Climate variability and cultural eutrophication at Walden Pond (Massachusetts, USA) during the last 1800 years

**DOI:** 10.1371/journal.pone.0191755

**Published:** 2018-04-04

**Authors:** J. Curt Stager, Brendan Wiltse, J. Bradford Hubeny, Eric Yankowsky, David Nardelli, Richard Primack

**Affiliations:** 1 Natural Sciences, Paul Smith's College, Paul Smiths, NY, United States of America; 2 Ausable River Association, Wilmington, NY, United States of America; 3 Department of Geological Sciences, Salem State University, Salem, MA, United States of America; 4 Department of Biology, Boston University, Boston, MA, United States of America; University of Hyogo, JAPAN

## Abstract

Recent shifts in the ecological condition of Walden Pond, MA, are of potentially wide interest due to the lake's importance as a cultural, historical, and recreational resource in addition to its scientific value as an indicator of local and global environmental change. Algal microfossils in six sediment cores document changes in hydroclimate and trophic status of the lake during the last 1800 years and extend two previous sediment core records of shorter length. Low percentages of planktonic diatoms in the longest cores (WAL-3, WAL-15) indicate shallowing and/or greater water clarity associated with a relatively arid interval during the Medieval Climate Anomaly, ca. A.D. 1150–1300, Cultural eutrophication of the lake since the A.D. 1920s caused diatoms in the genera *Asterionella* and *Synedra* to increase in relative abundance at the expense of *Cyclotella*, *Discostella*, and the chrysophyte alga *Mallomonas allorgei*. Percentages of *Asterionella* and *Synedra* have remained fairly stable since A.D. 2000 when a previous sediment core study was conducted, but scaled chrysophytes have become more numerous. These findings suggest that, although mitigation efforts have curtailed anthropogenic nutrient inputs to Walden Pond, the lake has not returned to the pre-impact condition described by Henry David Thoreau and may become increasingly vulnerable to further changes in water quality in a warmer and possibly wetter future.

## Introduction

Best known as an inspiration for Henry David Thoreau's *Walden*: *or Life in the Woods* [1] during the mid-19^th^ century, Walden Pond (Concord, MA) is also a heavily used recreational venue for hundreds of thousands of bathers, anglers, and visitors to the Thoreau cabin site annually [2]. During the early 20^th^ century, water clarity declined significantly due to a combination of factors including shoreline development and inputs of human wastes [2],[3]. Poisoning of the lake in 1968 with the organic pesticide, rotenone, repeated stocking with non-native sport fish, and regional climatic warming have also influenced the physical and biotic condition of the lake [2–4]. In recent decades, closure of a nearby town dump, shoreline stabilization, and upgrading of septic facilities by the management of Walden Pond State Reservation have mitigated some of the changes, but relatively few long-term measurements of water clarity, lake levels, or other limnological parameters are available with which to assess the success of such efforts [3–5]. Sediment cores, which can provide long, continuous records of phytoplankton community structure and other features of the ecosystem, are therefore an important source of long-term perspectives on environmental conditions within the lake. In this study, our analyses focus on the remains of diatoms and chrysophytes, which are microscopic plant-like algae that produce glassy, decay-resistant shells (known as "frustules" or "valves"), cysts, or scales that are often well preseved in lake sediments. Their remains possess diagnostic features that allow the identification of species which, in turn, aids in the development of inferences about past chemistry, water clarity, and other features of the lake based upon the known habitat preferences of individual taxa.

Two previous studies of sediment cores from the deepest basin in Walden Pond yielded 600-year [6] and ca. 1600-year records of pollen, microalgae, and geochemistry [7]. Both contained evidence of cultural eutrophication since the early 20^th^ century as indicated by shifts in the carbon and nitrogen isotope composition of the sediments and by pronounced increases of species within the genera *Asterionella* and *Synedra* that are often associated with enhanced phytoplankton productivity and reduced water clarity.

In this paper, we present diatom, chrysophyte, and geochemical records from six additional cores that help to document the trophic status and phytoplankton community of Walden Pond since A.D. 2000, when the last coring study was conducted. We also use the diatom records of our two longest cores to derive for the first time a qualitative reconstruction of late Holocene hydroclimate variability in the watershed, which provides new insights into the climate history of the northeastern United States for which relatively few such records are available.

## Study site

**Fig 1:**
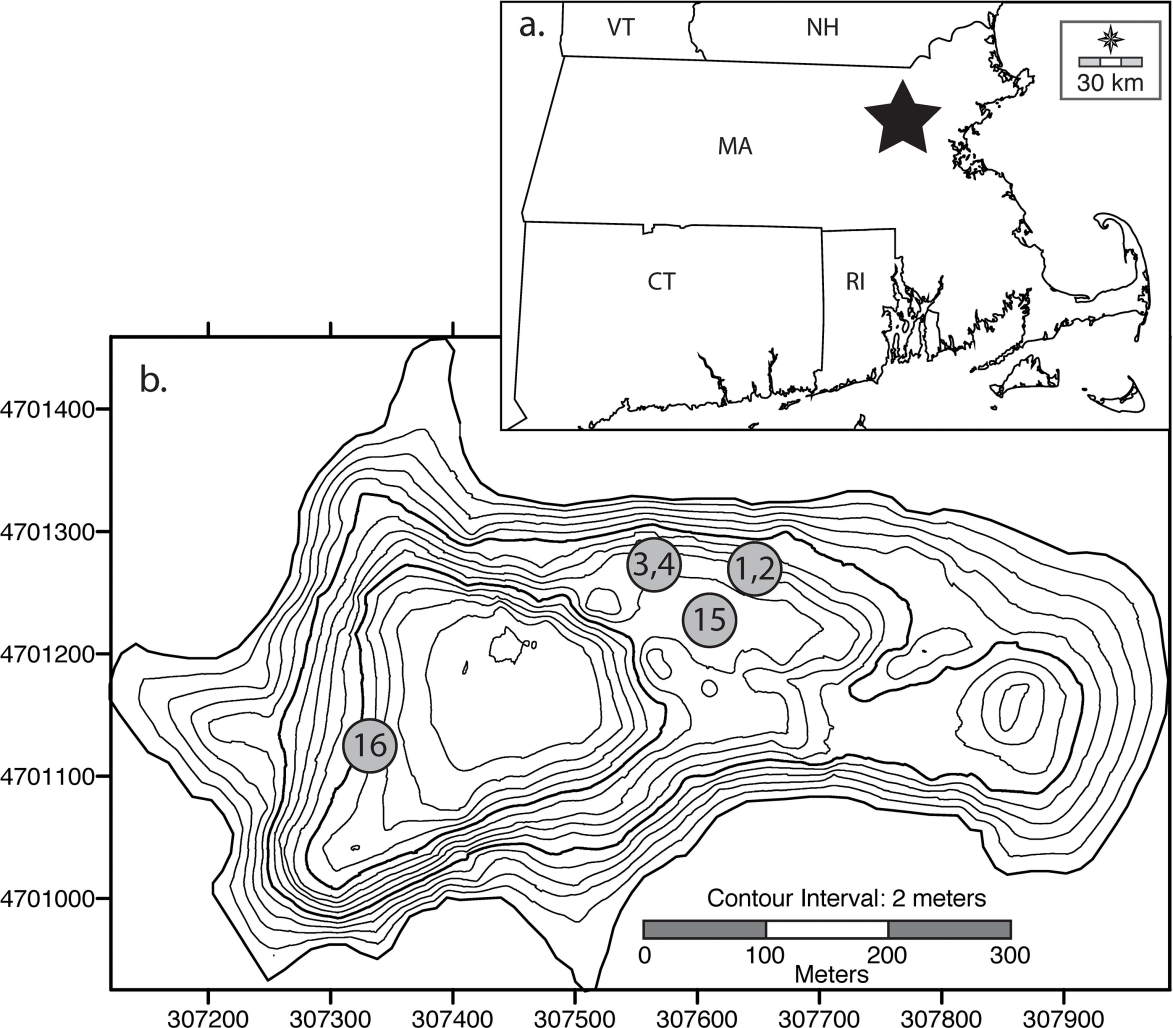
Location maps. (a) Southern New England, with the location of Walden Pond indicated by a star. (b) Walden Pond bathymetry with coring sites indicated. Numbers correspond to the numbers of the "WAL" cores discussed in the text. Regional map from http://d-maps.com/carte.php?num_car=7175&lang=en under a CC BY license, with permission from d-maps.com, original copyright 2007.

Walden Pond (42°26.3'N, 71°20.4'W; Fig 1) occupies a glacial kettle depression that formed in outwash sands and gravels atop granitic bedrock after the retreat of the Laurentide ice sheet [8]. It contains three basins of ca. 31 m, 20 m, and 18 m depth, depending upon the water level, which can vary over several meters between years, and it is the deepest natural lake in Massachusetts (Fig 1) [3]. Walden is a flow-through seepage lake with no surface inlet or outlet, an outcrop of the water table that receives about half of its volume from westward-flowing groundwater and the rest from direct precipitation [3]. Field observations showed that the hypolimnion (lower layer of the water column) was moderately oxygenated in A.D. 1939 (4 mg/L) [5] but more recently dissolved oxygen concentrations below 15 m depth have been typically at or close to 0 mg/L by the end of the summer stratification period [3]. Secchi depth, a measure of water transparency, was reportedly within the 6–9 m range during the 19^th^ century [1] and early 20^th^ century [5], but more often within the lower end of the 2.5–9 m range in recent decades [7]. Conductivity values are low (65–93 μS/cm) [7] and pH varies from ca. 6.5 during the spring mixing period to ca. 8.5 at intermediate depths in summer. The higher pH values have been attributed in part to productivity by benthic meadows of the fibrous, green macroalga, *Nitella*, that cover much of the lake bed between 6 and 13 m depths with a biomass 17 times that of the phytoplankton [3].

Concord was settled by British colonists in A.D. 1635, and the forests surrounding Walden Pond were logged to an increasing extent over the next three centuries, during which time oak (*Quercus*) became less common in the mixed woodland assemblage relative to birch (*Betula*) and pine (*Pinus*) [7]. A sequence of more recent human activities in the vicinity of Walden Pond, documented by Maynard [2] and summarized here, includes the building of a temporary shantytown close to the western shore in 1843 for workers during construction of the Fitchburg Railroad, which lies close to the western shore and has been in operation since 1844. Sparks from steam engines frequently caused forest fires near the lake during the 19^th^ century, including exceptionally large fires in 1894 and 1896. Wood-cutting operations left hill-slopes along the eastern and northern shores partially deforested in 1840–1841, 1851–1852, and 1918, and high water levels killed many lake-side trees during the early 1850s. A commercial picnic area and beach were operated on the western shore during the 1860s through the 1890s, but were demolished in 1903. As public usage of the lake increased further, underbrush and trees were cleared for walking along the water's edge and forest fires became more frequent.

After Walden Pond State Reservation was established in 1922, beach and bath-house facilities were constructed on the eastern shore. By the early 1930s, hundreds of thousands of swimmers used the facility in summer, and a new footpath to Thoreau's cabin site beside a cove on the northwestern shore caused large amounts of soil to wash into the lake, as did deforestation, landscaping, and enlargement of the beach by county commissioners in 1957. More than half of the summer phosphorus budget of the lake may now be attributable to urine released by swimmers [3]. Stocking of the lake with game fish was recorded as early as 1875, and it has been frequently stocked with rainbow trout (*Oncorhynchus mykiss*) and brown trout (*Salmo trutta*) in the years since it was treated with rotenone in 1968. The Reservation has been managed since 1975 by the Massachusetts Department of Conservation and Recreation, during which time shoreline stabilization and restoration efforts have reduced soil erosion, and closure of the nearby Concord landfill during the early 1990s reduced the prevalence of waste-deposition by gulls on the lake [2],[3].

## Materials and methods

Field permits for sampling in Walden Pond were provided by the Massachusetts Department of Conservation and Recreation. In June, 2015, two cores (WAL-1, WAL-4; 25 cm lengths) were collected with a UWITECH gravity corer from 15 m depth in the central basin, where two gravity-driven piston cores (WAL-2, WAL-3; 114 cm and 84 cm lengths, respectively) were also collected with a modified Kullenberg corer (Fig 1). Previous studies had focused on the deep western basin, but the shallower central basin was selected for this investigation in order to provide supporting information from that understudied sector of the lake. Another reason for this choice was the likelihood that benthic diatoms associated with shallower microhabitats would be more strongly represented in that location, thereby providing more robust time series of the ratios of planktonic to benthic taxa that can reflect variability in lake levels and hydroclimate [9–12].

As photosynthetic algae, benthic diatoms require sunlight, and their distribution on a lake bed is therefore influenced by related factors including water depth, clarity, and the proximity of littoral habitats [13]. Higher (lower) percentages of benthic (planktonic) taxa in a core, for example, can reflect increased light penetration to the bottom due to the lowering of lake levels, reduced inputs of dissolved organic carbon, a lateral shift of littoral habitats closer to the coring site, or a decreased influx of nutrients that support planktonic productivity, all of which are likely to occur during prolonged droughts [9–14]. Variations in the relative abundances of planktonic diatoms (referred to here as %PLANK) within the longer WAL-3 and WAL-15 cores are here taken to represent qualitative changes in effective moisture, with decreases of %PLANK representing generally drier hydroclimatic conditions that tend to favor the growth of benthic taxa through changes in light penetration to the bottom. This interpretation is supported by statistically significant relationships between modern-day water depth and %PLANK in the surface sediments of Walden Pond (this study) and other North American lakes [9–12].

Care was taken to avoid sites where *Nitella* mats interfered with penetration of the core barrels into the sediment (Fig 1), and one preliminary core was rejected in the field due to partial clogging with *Nitella*. Twelve samples of the mud-water interface were also collected from the central basin at 2 to 19 m depths, and a second field crew collected a gravity core (WAL-15; 72 cm length) from 19 m depth near the middle of the central basin (Fig 1). In 2016, a gravity core (WAL-16; 31 cm length) was collected from ca. 24 m depth in the deep western basin in order to better document recent changes in the phytoplankton community with cores from different sectors of the lake (Fig 1). All but one of the cores were extruded vertically in the field, subsampled in 0.5–1.0 cm increments, and stored in plastic sample bags under refrigeration. For WAL-15, the mud-water interface was stabilized with a plug for transport, and the core was split longitudinally for subsampling.

Radiocarbon ages of oven-dried organic sediments and sieved pollen fractions were obtained for 14 samples in the long cores WAL-2, WAL-3, and WAL-15 (Table 1), and calibrations were determined with CALIB version 7.1.0 (IntCal13) [15]. For WAL-3, ^137^Cs and ^210^Pb analyses on dried, powdered sediments were also performed by staff at the Saint Croix Watershed Research Station (MN, USA).

**Table 1:** Radiocarbon ages and calibrated age ranges of sediment samples from Walden Pond cores WAL-2, WAL-3, and WAL-15. The radiocarbon ages for WAL-2 denoted by "X" were not calibrated due to evidence of sediment disturbance (see text).

Depth (cm)	^14^C age (yr BP)	Calibrated Year A.D. 2-sigma (*prob*.)	Sample #
**WAL-2**			
27.5	1990 ± 30	x	134219
36.5	485 ± 30	x	134218
56.5	380 ± 2	x	134217
75.5	485 ± 35	x	134216
100.5	1330 ± 15	x	134215
111.5	1530 ± 20	x	131316
**WAL-3**			
40.5	345 ± 15	1472–1527 (0.40) 1554–1633 (0.60)	134223
51.5	490 ± 15	1416–1441	134222
69.5	1080 ± 15	899–923 (0.23) 946–1013 (0.77)	134221
78.5	1360 ± 20	645–678	134220
**WAL-15**			
35.5	535 ± 25	1322–1347 (0.19) 1392–1435 (0.81)	142334
47.5	980 ± 15	1017–1046 (0.72) 1090–1122 (0.24) 1139–1148 (0.04)	142335
57.5	1290 ± 20	668–726 (0.64) 738–768 (0.36)	142336
64.5	1660 ± 25	266–271 (0.01) 332–426 (0.99)	142337

**Fig 2:**
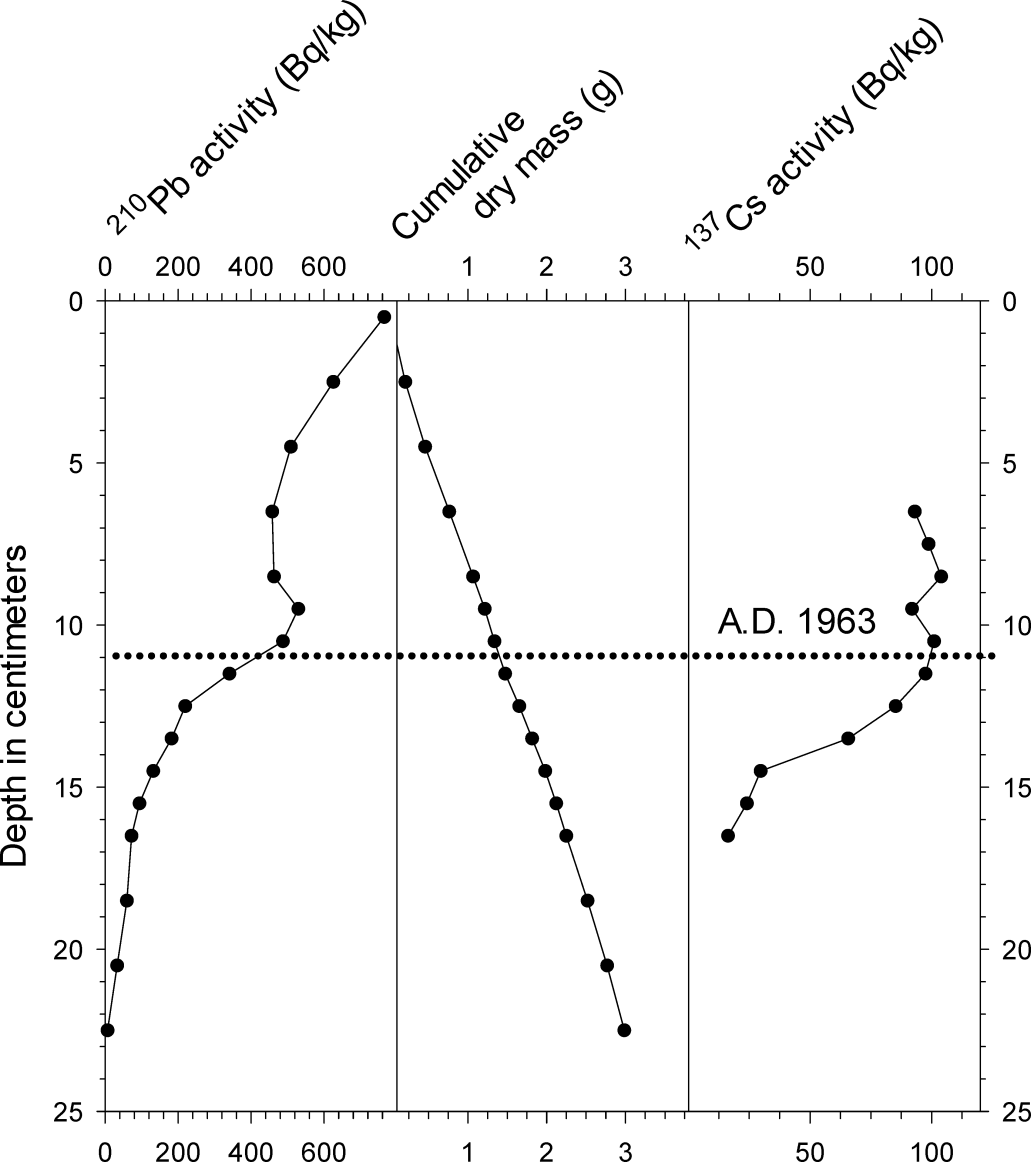
Lead-210 activity, cumulative dry mass, and ^137^Cs activity profiles from core WAL-3. Horizontal dotted line indicates the ca. A.D. 1963 interval as inferred from the ^210^Pb and ^137^Cs data.

**Fig 3:**
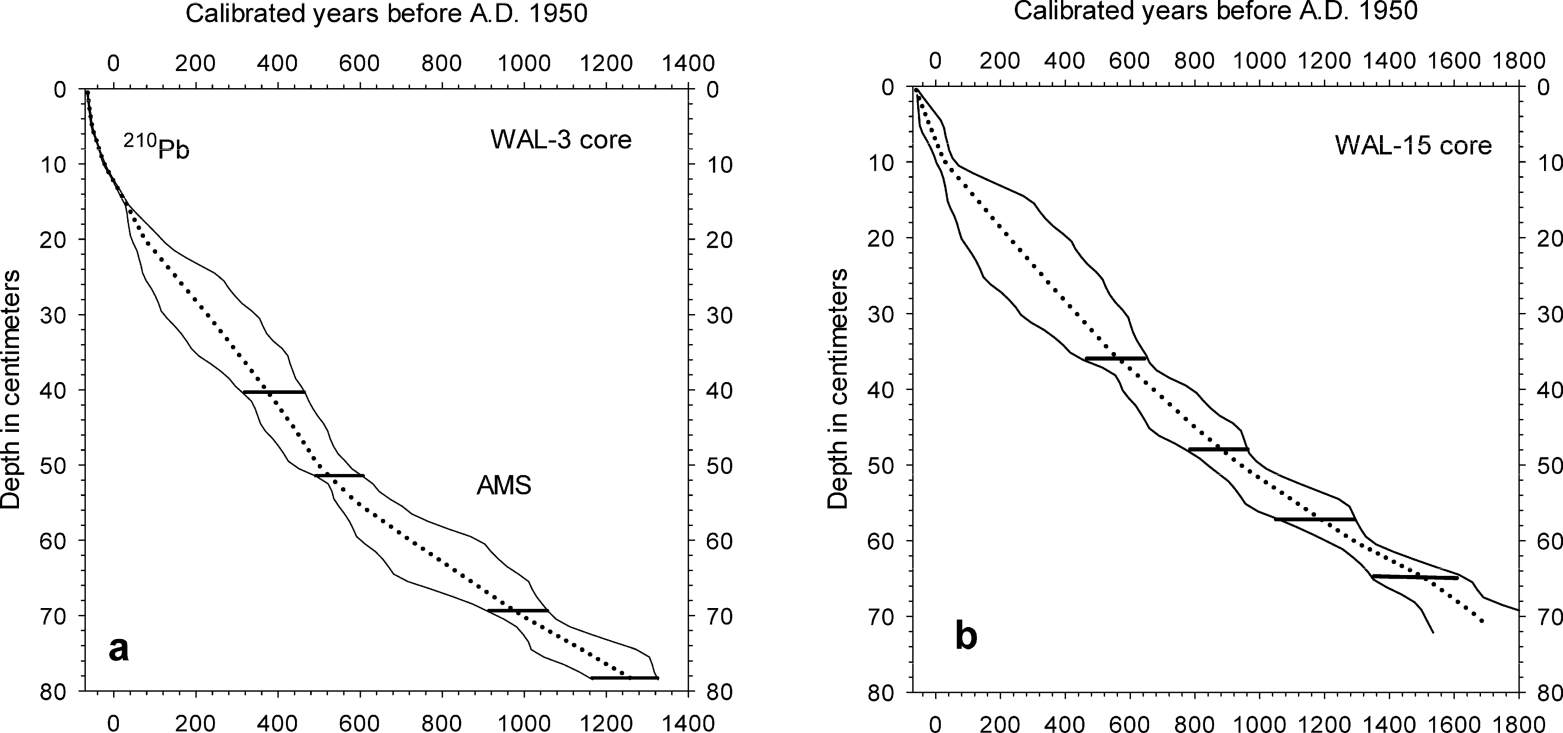
Age-depth relationships for Walden Pond cores WAL-3 and WAL-15. Solid lines represent the upper and lower 95% confidence intervals of the BACON age models. Dotted lines indicate the "best" model based on the weighted mean ages. Horizontal bars represent calibrated radiocarbon ages. Note the slightly different age scales in the two charts.

An age model for core WAL-3 was constructed from the ^137^Cs profile and the ^210^Pb activity profile using the constant rate of supply method [16], in combination with four accelerator mass spectrometry (AMS) dates on pollen fractions (Table 1, Figs 2 and 3). Four radiocarbon ages on bulk organic sediment were used to develop an age model for WAL-15 (Table 1, Fig 3). Radiocarbon ages were not obtained for the shorter cores due to cost constraints, which also prohibited additional ^210^Pb and ^137^Cs analyses for cores other than WAL-3. Major changes in their microfossil stratigraphies were instead used for comparison with the longer records for which radiometric dates were available.

Age-depth relationships were constructed using Bayesian age modeling on ^210^Pb and AMS data for core WAL-3 and on radiocarbon data alone for WAL-15 with the BACON version 2.2 modeling package in R (Fig 3) [17]. For the WAL-3 core, ^210^Pb dates were input into the model as uncalibrated dates with their appropriate error ranges as established by the constant rate of supply model. Pronounced inconsistencies in the radiocarbon ages of samples from core WAL-2 (Table 1) made age model construction and detailed microfossil analyses for that core unfeasible.

Because of the delicate nature of the microfossils and the disaggregated nature of the sediments, and because no evidence of extensive clumping or organic coatings was found in wet-mounted diatom samples, no chemical processing was used in the preparation of microscope slides for diatom and chrysophyte analyses. Subsamples were dried on glass coverslips and mounted on glass slides with Permount^TM^ mounting medium. Diatom valves and chrysophyte scales and cysts were enumerated at 1000X under oil immersion, using standard references for identification [18–21]. At least 500 diatom valves per sample were identified to species level in all cores at varying increments of 1–10 cm in order to characterize the general nature of the algal community during the last 18 centuries. The common planktonic diatom *Cyclotella bodanica* has recently been classified elsewhere as *Lindavia bodanica*, *Handmannia bodanica*, and *Puncticulata bodanica* [22], but we refer to it here as *C*. *bodanica* sensu stricto for the sake of consistency with previously published records [7].

Sample intervals of 1 cm were employed throughout the long cores WAL-3 and WAL-15 in order to infer paleohydrological conditions from the relative abundances of planktonic and benthic diatoms as determined from counts of at least 500 diatom valves per sample. The most abundant diatom taxa were also enumerated to species level at 1 cm increments within the uppermost 20 cm of cores WAL-1, WAL-3, WAL-4, WAL-15, and WAL-16, in order to examine the consistency of changes in the diatom community at multiple locations and thereby to better evaluate the degree to which the more rigorously dated record of WAL-3 represents the history of Walden Pond.

Changes in sediment organic content, as estimated by percent weight loss on ignition (%LOI) at 550°C [23],[24], was measured in 1 cm increments in all of the cores except for WAL-15, which was analyzed for total carbon using an Elementar Microcube elemental analyzer. Carbon values for WAL-15 were converted to organic matter (%LOI) estimations for comparative purposes by assuming that elemental carbon comprises 44% of total organic matter [24].

## Results

### Radiometric analyses

Activity of ^210^Pb in WAL-3 declined exponentially with depth with the exception of the 9–11 cm interval, which indicated physical disturbance of the sediments and/or a shift in deposition rates during the A.D. 1960s to A.D. 1970s (Fig 2). Background levels were reached in the ca. 22–23 cm depth interval (Fig 2). Activity of ^137^Cs in WAL-3 displayed a broad maximum between 10 and 12 cm that was taken to represent the ca. A.D. 1963 global peak in atmospheric thermonuclear bomb testing (Fig 2). The diffuse nature of the peak might represent post-depositional migration of ^137^Cs upward in the unconsolidated organic sediments [25],[26], and it makes the ^137^Cs data less reliable as a tool to test the accuracy of the ^210^Pb age model.

The age-depth profiles derived for WAL3 and WAL-15 displayed relatively little variability other than generally increasing age increments per centimeter with depth (Fig 3). The radiocarbon ages of six subsamples from WAL-2, however, displayed major age reversals that made the core unsuitable for detailed analysis (Table 1). The lack of such reversals or major irregularities in the age-depth profiles for WAL-3 and WAL-15 indicated that their stratigraphies were not significantly disturbed and that the bases of the cores were roughly 1500 and 1800 calibrated radiocarbon years old, respectively (Table 1, Fig 3). BACON model iterations were stable in cores WAL-3 and WAL-15, and the prior distributions established for both accumulation rate and memory closely matched the posterior distributions [17].

### Microfossil analyses

**Fig 4:**
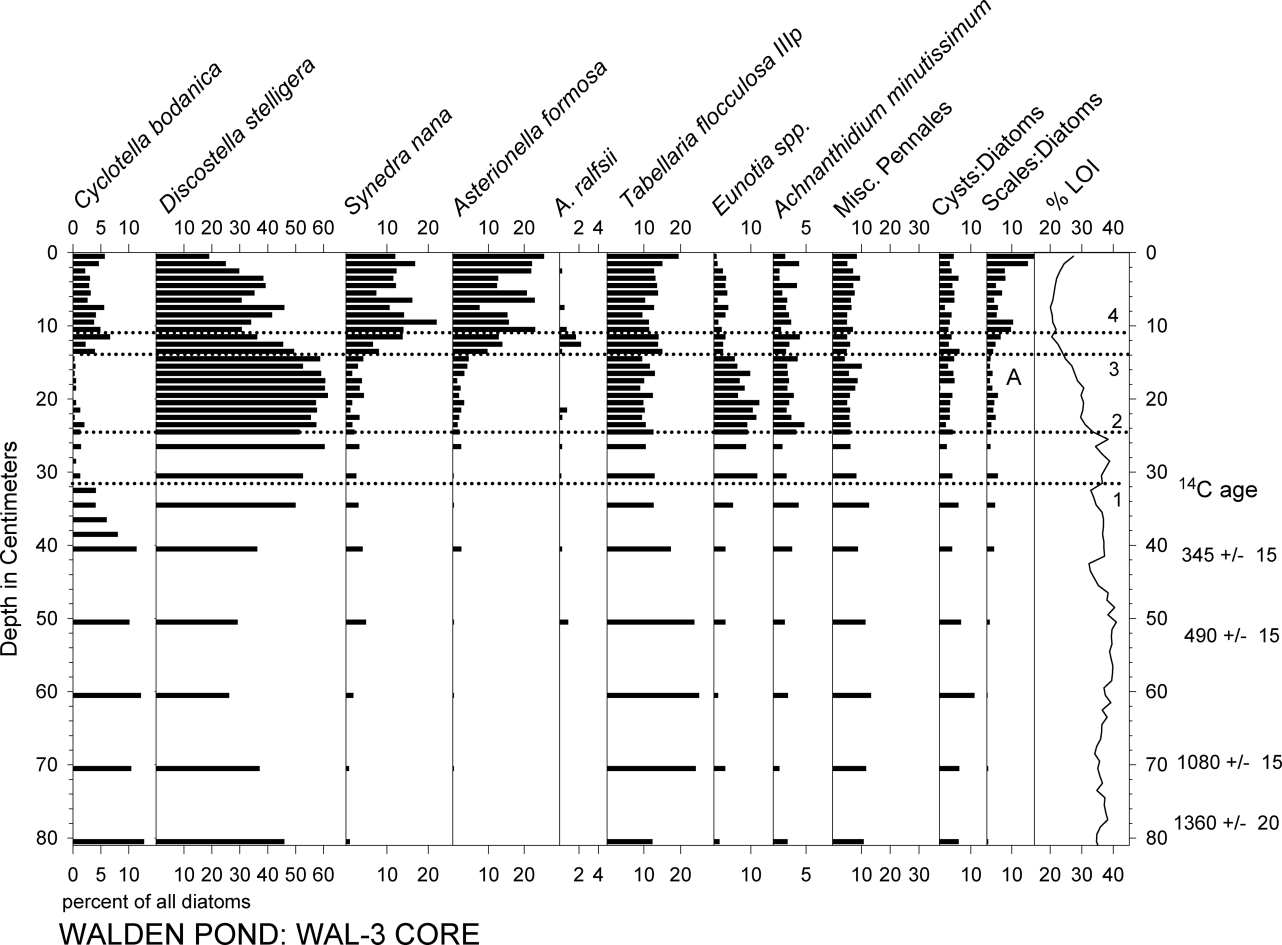
Percent abundances of the most common diatom taxa and %LOI in core WAL-3. Horizontal dotted lines: (1) Onset of low *Cyclotella* percentages. (2) Decrease in %LOI indicating reduced organic matter content in the sediments. (3) Onset of increased *Asterionella and Synedra* percentages. (4) First peak of chrysophyte scale abundances. Letter "A" indicates last occurrence of *Mallomonas allorgei*.

**Fig 5:**
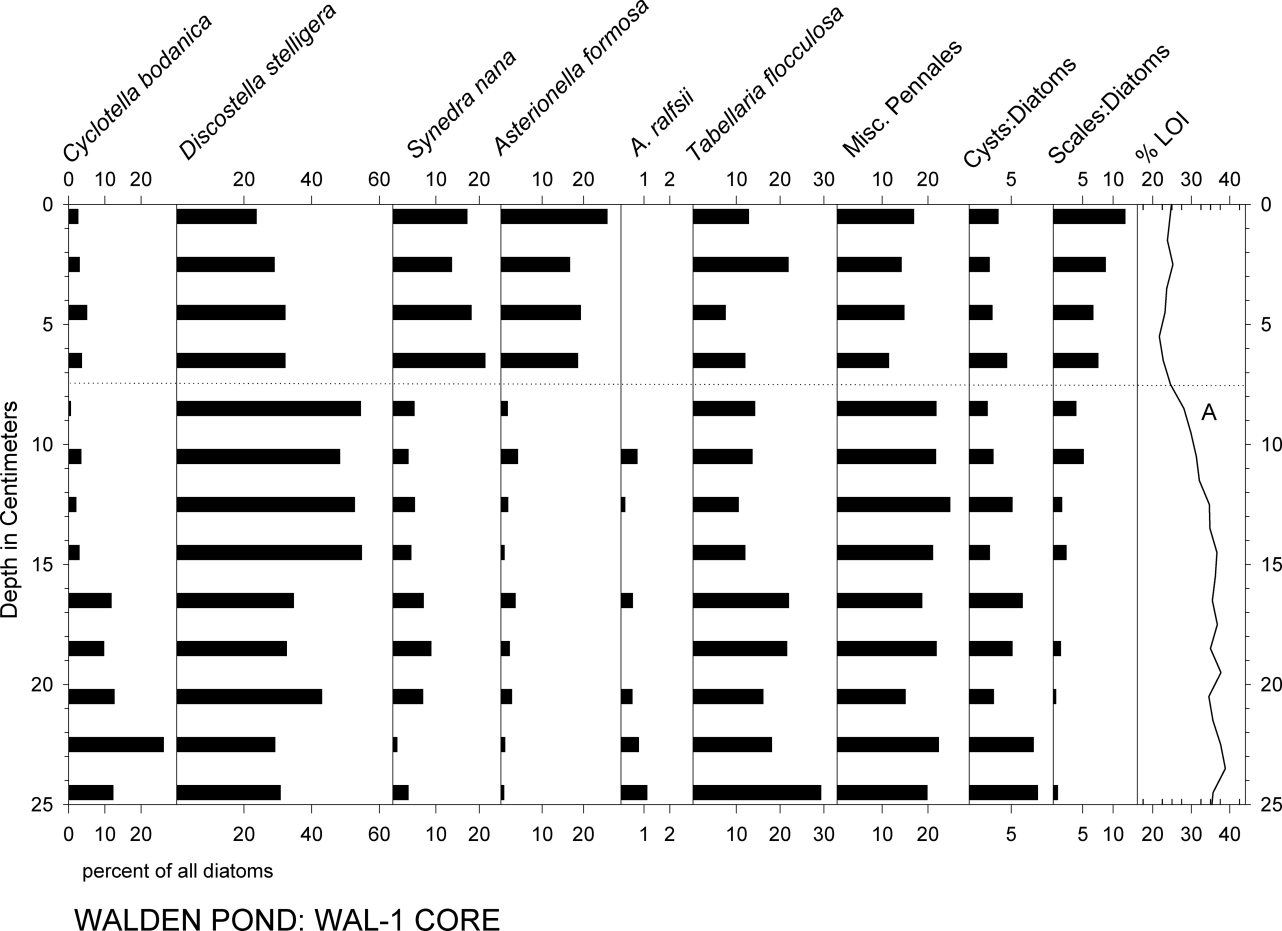
Percentages of the most common diatom taxa and %LOI in core WAL-1. Horizontal dotted line indicates transition to high percentages of *Asterionella* and *Synedra*.

**Fig 6:**
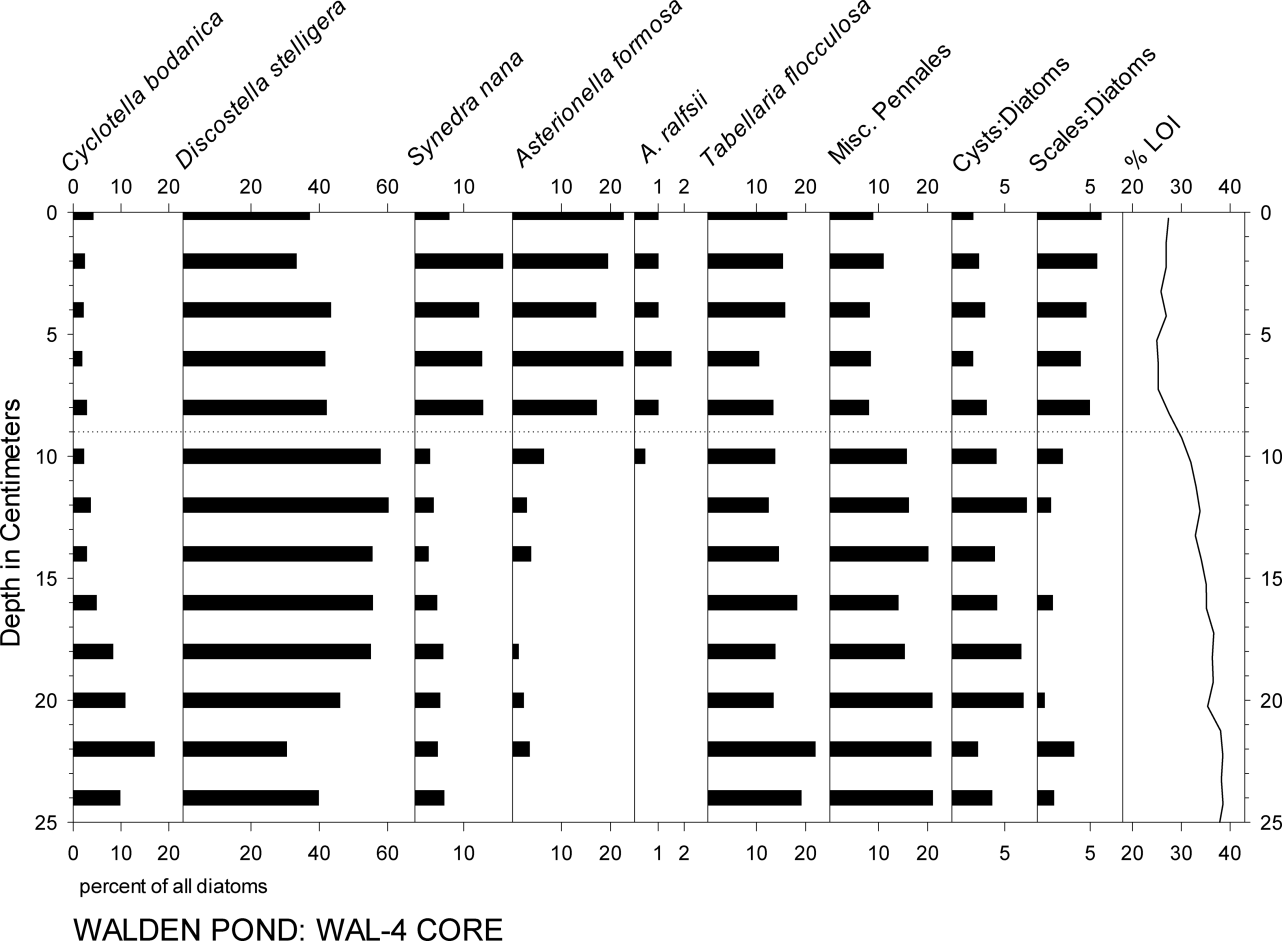
Percentages of the most common diatom taxa and %LOI in core WAL-4. Horizontal dotted line indicates transition to high percentages of *Asterionella* and *Synedra*.

**Fig 7:**
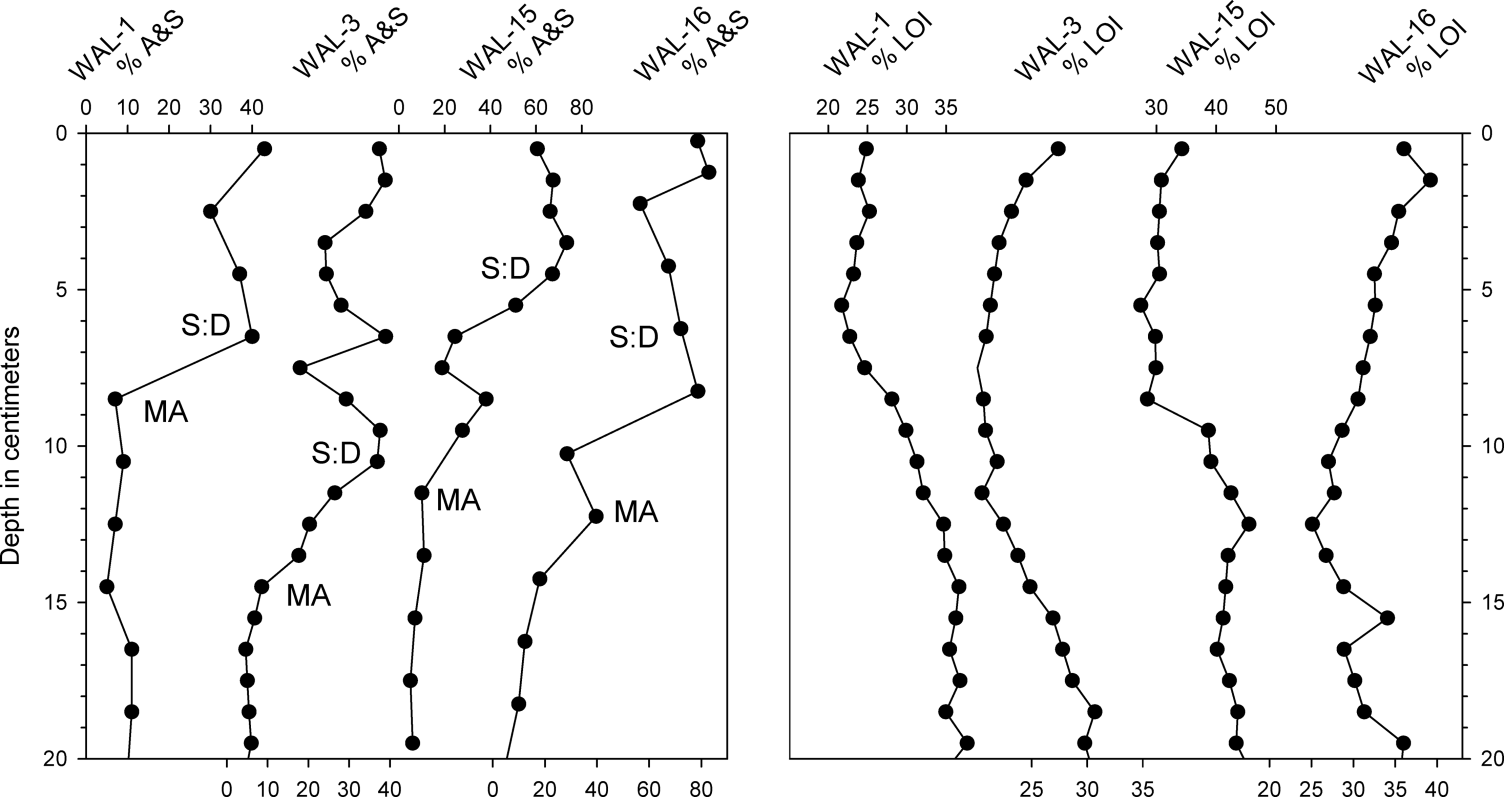
Comparison of microfossil and %LOI records in the uppermost 20 cm of four cores from Walden Pond. Left panel: Combined percentages of *Asterionella* and *Synedra* (A&S) were interpreted as representing cultural eutrophication during the last century. The most recent presence of the oligotrophic indicator species, *Mallomonas allorgei* in each core and a pronounced increase in the abundances of chrysophyte scales relative to diatoms are indicated by "MA" and "S:D," respectively. Right panel: Organic content in the core sediments as represented by weight loss on ignition (%LOI).

Below the 7–12 cm level in all of the cores, the most abundant diatom taxa were *Discostella stelligera*, *Cyclotella bodanica* sensu lato, and *Tabellaria flocculosa* var. IIIP, which together comprised roughly half to two thirds of the assemblages (Figs 4–6). In the younger sediments of all of the cores, *Asterionella formosa* and *Synedra nana* were abundant, as well (Figs 4–7). In WAL-3, for example, a rise in *A*. *formosa* to ca. 10–20% occurred above the 14 cm level, followed by a similar rise in *S*. *nana* (Fig 4). *Asterionella ralfsii* was rare in all cores, but represented up to 2% of the assemblage within the 10–13 cm interval in WAL-3 (Fig 4). Percentages of *C*. *bodanica* reached minimal values within the 5–15 cm interval of WAL-3, then rose again within the uppermost 5 cm of the core (Fig 4). A similar pattern of recent decline and recovery in *C*. *bodanica* was found in the other cores, as well, with the exception of WAL-4 which displayed no recovery at the core top (Fig 6).

**Fig 8:**
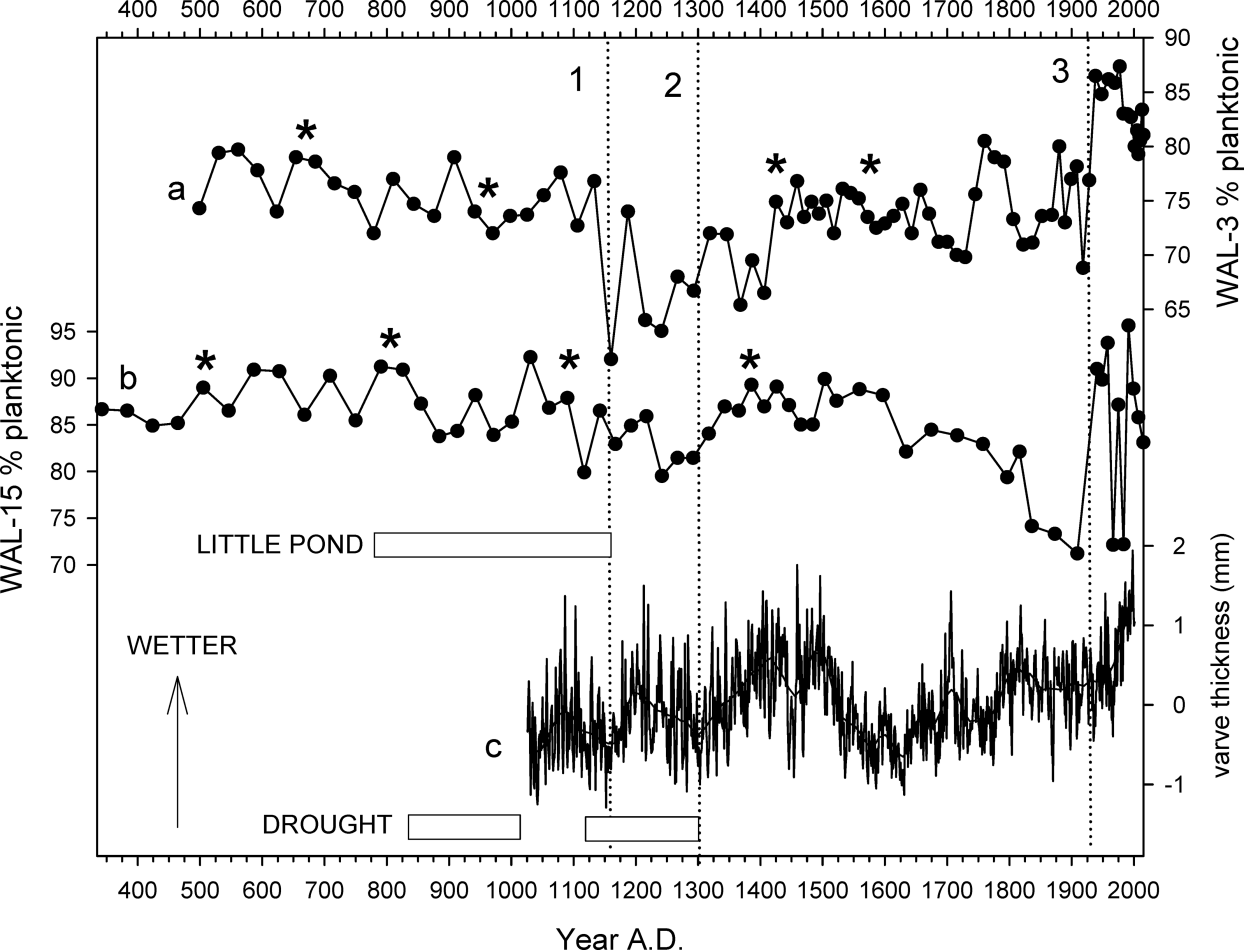
Comparison of sediment core records representing hydroclimate at Walden Pond and elsewhere in the northeastern United States. a, b. Percent planktonic diatoms in Walden Pond cores WAL-3 and WAL-15, respectively. Data after ca. A.D. 1930 primarily represent increased cultural eutrophication rather than climate alone. c. Composite hydroclimate record derived from varved sediments of a lake and estuary in New York and Massachusetts, respectively [38]. Horizontal bars represent drought periods at Little Pond, MA (upper)[36] and the New England region (lower)[34]. Vertical dotted lines #1–2 bracket the A.D. 1150–1300 interval, line #3 indicates approximate onset of cultural eutrophication. Asterisks indicate core-intervals with calibrated radiocarbon ages.

Percentages of diatom taxa that are typically found in planktonic habitats (%PLANK) fluctuated mainly within the 70–80% range in WAL-3, with minimal %PLANK in the 52–63 cm interval and maximal values in the most recent sediments above the 14 cm interval (Figs 4 and 8). In WAL-15, %PLANK typically ranged between ca. 80% and 90% in the lower portion of the core, with minimal values within the 42–50 cm interval and maximal values above the 10 cm interval (Fig 8). Percentages of planktonic diatoms were highest in the upper ca. 20 cm of WAL-1 (Fig 5), WAL-3 (Fig 4), WAL-4 (Fig 6), and WAL-15 (Fig 8), and exceptionally high in the upper half of deep-water core WAL-16. Percentages of planktonic taxa also increased significantly with depth in the surface sediment transect (r^2^ = 0.86, P<0.0001).

In all of the cores, abundances of chrysophyte scales relative to diatoms (S:D) were highest within the uppermost 10–12 cm (Figs 4–6). Common chrysophyte taxa in those recent assemblages included *M*. *crassisquama*, *M*. *elongata*, and *M*. *caudate*. In cores WAL-3, WAL-15, and WAL-16, S:D values approximately doubled again within the uppermost 4 cm (Fig 4), but they did not do so in WAL-1 or WAL-4 (Figs 5 and 6). Scales of *M*. *allorgei* were common in the older sediments but were rare to absent in sediments that were deposited after the rise in *Asterionella* and *Synedra* (7–12 cm range; Figs 3 and 7).

### Geochemical analyses

Profiles of organic matter as represented by %LOI revealed a decline of varying magnitude and duration within the uppermost sediments of all cores (Figs 4–7). In WAL-3, %LOI values declined above the 24 cm level before rising to intermediate values at the top (Fig 4). In most of the other cores, %LOI also declined and then increased within the uppermost sediments (Fig 7) but it did not rise again in WAL-1 or WAL-4 (Figs 5–7). Core WAL-15, from a deeper part of the central basin, yielded higher %LOI values, on average, than the cores from shallower sites that were situated closer to shore (WAL-1, WAL-3, WAL-4), and the highest %LOI values were found in core WAL-16 from the deep western basin (Figs 1 and 7).

## Discussion

### Consistency among the microfossil records

%LOI In general, the diatom stratigraphies of our cores were similar to those reported by Winkler [6] and Köster et al., [7], and they confirm that major changes in the phytoplankton community occurred during the 20^th^ century. All of the cores collected thus far from Walden Pond have recorded a pronounced increase in the prevalence of *A*. *formosa* and *S*. *nana* (A&S) after the A.D. 1920s, and a large, sustained increase in the abundance of chrysophyte scales during the late 20^th^ century.

**Fig 9:**
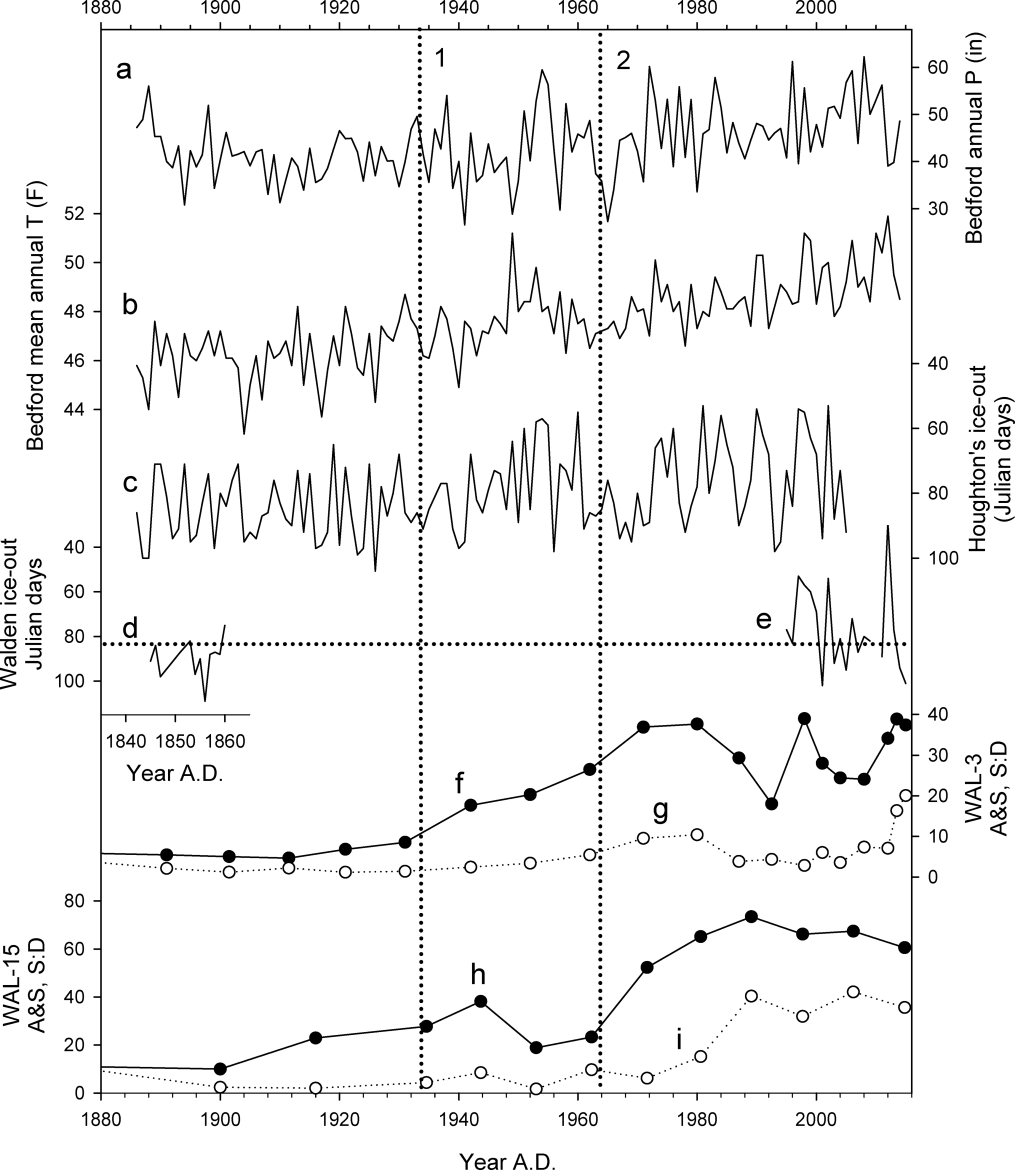
Comparison of temperature and precipitation in eastern Massachusetts since A.D. 1880 with ice-out and microfossil records from Walden Pond. (a, b) Precipitation and temperature, respectively, at Bedford, MA. (c) Ice-out dates at Houghton's Pond, MA. [47] (d,e) Spring ice-out dates at Walden Pond recorded by Thoreau [1],[4],[48] and Primack [4], respectively. (f, h) Percentages of *Asterionella* and *Synedra* (A&S) in cores WAL-3 and WAL-15, respectively. (g,h) Ratios of chrysophyte scales to diatoms (S:D) in cores WAL-3 and WAL-15, respectively. Horizontal dotted line indicates mean ice-out date at Walden Pond since A.D. 1880. Vertical dotted lines (1,2) indicate approximate onset of main increase in A&S percentages in cores WAL-3 and WAL-15, respectively.

The WAL-15 diatom record appears to indicate a slightly later increase in the eutrophication-indicators A&S than that of WAL-3 and the core analyzed by Köster et al. [7] (Fig 9). However, its age-depth model lacks support from the kinds of ^210^Pb and ^137^Cs profiles that were available for WAL-3, and we therefore interpret the timing of the recent microfossil changes in WAL-15 with greater caution.

The relative abundances of *C*. *bodanica* declined to near zero across the A&S transition in all cores, including that of Köster et al. [7], but the timing of the onset of the decline was not consistent. Köster et al. [7] dated the change to the A.D. 1600s, but in the WAL-3 and WAL-15 records it apparently occurred during the 17^th^ and 18^th^ centuries, respectively (Fig 4, WAL-15). Percentages of *C*. *bodanica* rose again within the A.D. 1960s intervals of the Köster et al. [7] core, WAL-3, and WAL-15, but not in WAL-1 or WAL-4. In addition, %PLANK values declined within the A.D. 1600–1900 interval of the WAL-15 record but not in WAL-3 (Fig 8). The reasons for these stratigraphic inconsistencies are unclear, but they show that single cores do not necessarily reflect the history of an entire lake with perfect precision.

Although the age-depth relationships reported by Köster et al. [7] are consistent with those in our own cores, the single radiocarbon age of 530 +/- 70 yr BP obtained by Winkler [6] for a sediment sample from 75–81 cm depth in her deep-basin core is much younger than those determined for corresponding intervals in WAL-3 and WAL-15 (Table 1, Fig 3) and the Köster et al. [7] core. The age model for the microfossil records reported by Winkler [6] might therefore require re-evaluation.

The lack of rising values of %LOI and % *C*. *bodanica* along with the presence of rare but distinctive *A*. *ralfsii* and *M*. *allorgei* at the top of core WAL-4 suggest that the upper section of its sediment record was missing and/or physically disturbed. As was the case with problematic radiometric age discrepancies in core WAL-2, the causes of such stratigraphic irregularities cannot be identified with certainty but could include disturbances by boat anchors, contact with the bottom by free-divers, or burrowing by over-wintering turtles which are common in the pond.

The upper sediments of WAL-15 (0–18 cm) and WAL-16 (0–14 cm) were dark brown to black, much darker than the uniformly brown sediments below, but there was little to no apparent color variability in the other cores collected for this study. This lack of darkening is likely due to higher concentrations of dissolved oxygen at their shallower collection sites which lie closer to the upper depth limit of summer hypoxia in the lake and the lower depth limit of oxygen-producing *Nitella* meadows [3].

### Hydroclimate history

Climatic interpretation of %PLANK in sediment cores can be problematic where anthropogenic soil erosion and nutrient-loading also influence diatom communities [7],[12]. However, historical and paleolimnological records indicate relatively little human impact on Walden Pond before the late 19^th^ century [1–7]. We therefore interpret %PLANK in the older portions of the two longest cores as a qualitative indicator of hydroclimate variability before the late 19^th^ century.

We focus here on an interval of reduced %PLANK within the WAL-3 and WAL-15 cores (Fig 8) that appears to represent a period of reduced effective moisture ca. A.D. 1150–1300 during the Medieval Climate Anomaly (MCA; A.D. 950–1250) [27]. The WAL-3 record also includes a secondary decline that might extend the period to ca. A.D. 1420, but it is less pronounced in WAL-15 (Fig 8). WAL-3 was collected from a site that was both closer to shore and shallower than that of WAL-15, which is probably why its pre-19^th^ century sediments contain higher percentages of benthic diatoms than WAL-15 and greater variability, as well (ranges 20–40% and 8–20%, respectively). Köster et al. [7] did not provide data on benthic taxa in their deep-water core that could be compared to our own records, but the relatively low percentages of benthic taxa in WAL-16 (ca. 5–10%) also suggest that diatom assemblages in the deeper sectors of the lake may be less sensitive indicators of hydroclimate variability and support our choice of coring sites in the shallower central basin.

Other paleoecological records have documented aridity in the northeastern United States during the MCA [28], although estimates of its timing and duration vary. Drought conditions were inferred from pollen and charcoal data for the lower Hudson Valley, NY, around A.D. 800–1300 [29], and Sidney Bog, ME, registered relatively dry conditions ca. A.D. 1050–1400 [30]. Dry conditions ca. A.D. 900–1400 have also been inferred from records in northwest Ontario [31], the Great Lakes region [32], and the southwestern United States [33],[34].

In contrast, lake level records from Deep, Davis, and New Long Ponds, MA, revealed no evidence of aridity during the MCA and instead placed a shorter period of low lake levels earlier, ca. A.D. 650–750 [35]. At Little Pond, MA, it is reported to have occurred from ca. A.D. 750–1150 as indicated by increased abundances of spores of the benthic plant *Isoëtes* and low sediment organic content (Fig 8) [36]. A high-resolution diatom record from Wolf Lake, NY, registered a low stand ca. A.D. 950–1100 but not A.D. 1150–1300 [12]. Dendroclimatological records registered severe droughts throughout much of North America A.D. 832–1074 and A.D. 1122–1299 (Fig 8) [37] but relatively little change in New England then, a conclusion that the Walden Pond records do not support. A composite time series based on varved sediment records from Rhode Island and New York indicates variable but mostly wetter conditions during the A.D. 1100–1300 interval (Fig 8)[38]. That increase in precipitation relative to evaporation, however, was not strongly registered in the New York record and could presumably represent a maritime microclimate in coastal Rhode Island rather than the region as a whole. The Walden core data lack sufficient temporal resolution to rigorously define the nature of decadal-scale variability during that period, but the WAL-3 diatom record more closely resembles the aforementioned varve record than WAL-15 (Fig 8).

At present, the limitations of radiocarbon dating and a need for more high-resolution paleolimnological records from New England make it difficult to determine whether these differences represent true regional variability or uncertainty in chronologies. Precipitation is spatially variable, particularly in complex terrain such as that which characterizes the northeastern United States, and lakes of different forms and hydrological settings can respond differently to it, so more records will be required before the nature of hydroclimate variability in this region during the late Holocene can be more fully characterized.

The large increase in %PLANK that occurred in cores WAL-3 and WAL-15 within the uppermost 10–13 cm (Figs 4–8) was more the result of cultural eutrophication than climate [3],[7], and is discussed below.

### Recent changes in the phytoplankton community

Phytoplankton productivity in Walden Pond has increased notably since the A.D. 1920s, following recreational development of the shoreline [2],[3],[5],[7]. Other recent nutrient inputs included erosion of sediment from the lakeshore and walking trails that probably also contributed to declines in %LOI within the cores through dilution with inorganic mineral grains (Fig 7)[2]. Mitigation measures such as stabilization of the shoreline and trails and improvement of public sanitary facilities have since restricted anthropogenic nutrient inputs to the lake, but their more recent effects on the lake remain to be evaluated with the aid of long-term perspectives provided by sediment cores.

The previous core studies at Walden Pond documented an abrupt decline in the abundance of benthic *Isoëtes* during the mid-20^th^ century, probably due to shading from enhanced planktonic productivity [6],[7]. Köster et al [7] also identified an ecological transition starting around A.D. 1930 in which *C*. *bodanica* and *D*. *stelligera* were ljoined by abundant *A*. *formosa* and *S*. *nana*, taxa that are commonly associated with cultural eutrophication in North American lakes [39–43]. That transition was followed by increased abundances of chrysophytes during the late 20^th^ century.

The diatom and chrysophyte records of our cores corroborate those previous studies and extend them forward in time to A.D. 2015. In WAL-1, WAL-3, WAL-15, and WAL-16, scales of the chrysophyte *M*. *allorgei* were present throughout the older sections of the cores but were not found above the A&S transition (Fig 7). This species is typically found in oligotrophic lakes [44] and its disappearance from the sediment assemblages probably reflects the eutrophication that is so prominently registered in the diatom records.

We further examine the microfossil records of Walden Pond here in qualitative terms in order to address two basic question about the condition of the lake in recent times: What caused the recent changes in the phytoplankton community, and has the lake been returning to its pre-impact condition or developing a new "no-analog" state?

Climatic warming can affect plankton communities by enhancing thermal stratification which, in turn, can amplify internal loading of sediment-bound nutrients through hypoxia at the bottom of the lake [45]. Increased precipitation can also increase nutrient inputs through erosion of nearby soils and lake-shore deposits [12],[46]. However, the rise in A&S during the early 20^th^ century predates by several decades the notable increases in temperature and precipitation that occurred in Massachusetts since the A.D. 1960s (Fig 9). No significant changes are evident in the duration of lake ice cover, an indirect indicator of regional warming, in records from Houghton's Pond, MA [47], or Walden Pond [4],[48] that could also help to explain changes in the microfossil records (Fig 9). We therefore concur with previous authors that recreational use of the lake was the primary trigger for eutrophication in Walden Pond [2],[6],[7], although climatic changes could also be contributing to plankton productivity in recent decades.

The darkening of sediments in the upper sections of cores WAL-15 and WAL-16 probably reflect a greater prevalence of reducing conditions in the hypolimnion within the last century or so because iron compounds in sediments exposed to low-oxygen environments are less likely to produce the pale, rusty coloration that is typical of oxidzed ferric iron. Such conditions could, in turn, reflect longer stratification seasons and/or increased phytoplankton productivity. The WAL-15 age model suggests that the color boundary corresponds to the mid-18^th^ century, but in WAL-16 the change occurred only slightly below the A&S transition of the early 20^th^ century. The lower boundaries of the darkened zones in those cores could reflect downward penetration of low-oxygen conditions in addition to deposition under hypoxia so they do not necessarily indicate precise timing of the onset of the change. Nonetheless, they do suggest that cultural eutrophication has likely caused cause dissolved oxygen concentrations in the hypolimnion to decline.

Comparison of A&S percentages in four cores (Fig 7) reveals no consistent, significant trends since the initial A&S transition. We interpret this to mean that cultural eutrophication in Walden Pond has more or less stabilized since the mid-20^th^ century. However, the cores also indicate that the phytoplankton community and dissolved oxygen conditions have not returned to their pre-impact condition. It is likely that Walden Pond remains in a state of enhanced productivity due to a combination of factors that, in addition to direct anthropogenic nutrient enrichment [3], could also include atmospheric nitrogen deposition [49],[50], surface warming [4],[50–53] and internal loading of nutrients from bottom sediments [43] in the deepest portions of the basin where it is too dark for *Nitella* meadows to oxygenate the surrounding water.

The recent increase in scaled chrysophytes in Walden Pond resembles similar increases in other temperate zone lakes that have been attributed to the competitive advantages that longer summer stratification seasons give to small, motile flagellated algae [53],[54]. However, its causes at Walden Pond are difficult to ascertain. Much of the increase in chrysophyte abundances in North American lakes has been among colonial taxa such as *Synura* [55],[56] rather than the unicellular *Mallomonas* that is most common in Walden Pond, and multiple local anthropogenic impacts on the lake during the last century also make it difficult to identify the cause.

Although the reasons for the recent rise in abundance of chrysophytes remain unclear, the longer cores demonstrate that the change is unprecedented during the last 1800 years at Walden Pond. It is not readily related to changes in regional precipitation, temperature, or lake ice-cover (Fig 9), and therefore seems likely to be more the result of increased nutrient inputs or trophic cascades than climatic factors. Regardless, the recent large increase in scaled chrysophytes, along with the persistence of A&S and the decline of *Isoëtes* and *M*. *allorgei*, also means that Walden Pond has not been restored to its pre-impact condition. Like many other lakes around the world [54], Walden Pond has instead moved to a new ecological state that is unique in the last 1800 years if not the entire post-glacial history of the lake.

Increasing %LOI within the uppermost intervals of WAL-3, WAL-15, and WAL-16 since the 1980s might represent reduced inorganic sediment inputs from stabilization of shorelines and foot trails, but it could also reflect greater primary productivity and/or declining oxygen concentrations in the hypolimnion. The lack of such an increase of %LOI at the top of core WAL-1 (Fig 7) most likely reflects the absence of the youngest sediments from that core.

### Lake management in a changing future

The widespread occurrence of low lake levels during the MCA has implications for the interpretation of climate model projections that anticipate rising temperatures and heavier precipitation in New England during this century [57], trends that have already become evident in Massachusetts in recent decades (Fig 9). The warm MCA was not caused by rising greenhouse gas concentrations as much of the warming of the last half-century was [58], but as an imperfect historical analog it suggests that lake levels might not rise significantly in a warmer future even if total annual precipitation increases. Less snowmelt from shorter winters, for example, could reduce spring and summer groundwater supplies, and higher temperatures could enhance evaporation, thereby reducing effective moisture.

Paleolimnological evidence such as that from Crawford Lake, Ontario [43],[59], demonstrates that the effects of cultural eutrophication can persist for centuries after the causal nutrient inputs have ceased, and that it is often easier to prevent eutrophication than to reverse it once it occurs. The sediment darkening and high percentages of A&S in the recent sediments of Walden Pond therefore indicate not only that the lake ecosystem is now quite different from that described by Thoreau [1],[48] but also that it may be primed for more severe reductions in water clarity in a warming future. Increasing temperatures during this century [57], for example, are likely to encourage heavier summer recreational use of the lake, and lengthening of the summer stratification season through warming of the lake surface could also further enhance internal nutrient loading [60].

Major reductions in the extent of *Nitella* meadows due to decreasing water transparency under such conditions could represent an ecological tipping point that could severely and permanently reduce the clarity of the lake and degrade its recreational value. However, the taxonomic composition and ecology of the aquatic macrophyte communities of Walden Pond have never been fully characterized. Although the benthic meadows have been described as being composed primarily of *Nitella* [3], Deevey [5] reported *Fontinalis* moss, not *Nitella*, being dredged from 15.7 m depths, and the decline of *Isoëtes* spores in sediments during the mid-20^th^ century [6],[7] suggests that *Nitella* might not have been as prevalent in the past as it is now. Considering the importance of benthic vegetation to the oxygen and nutrient dynamics in Walden Pond, such information could be useful in future management of the lake. For example, different species of *Nitella* and other benthic macrophytes can have different light regime requirements [61],[62], so changes in species composition within the benthic meadows could serve as biotic indicators of changing water clarity.

As Jeppeson, et al. [63] noted in a review of lake management practices in a changing climate, future warming and increased runoff from precipitation mean that nutrient inputs to many lakes must be reduced if they are to maintain the same ecological state they are in today. It will therefore be prudent to further reduce the flow of anthropogenic nutrients to Walden Pond under the warmer, wetter conditions that most climate models project for New England during the 21^st^ century [57]. Swimmers are probably the largest source of such nutrients now, and demand for the beach facilities is likely to increase in a warmer future. Swimmer-education programs or construction of a separate swimming pool facility nearby to relieve pressure on the lake might therefore be advisable.
